# Sclerosing Angiomatoid Nodular Transformation of the Spleen in a Patient With Granulomatosis With Polyangiitis

**DOI:** 10.7759/cureus.53907

**Published:** 2024-02-09

**Authors:** Jesus Delgado-de la Mora, Eduardo Martín-Nares, Gabriel Quintero-Bustos, Daniel Montante-Montes de Oca, Braulio Martínez Benitez

**Affiliations:** 1 Department of Pathology, Weill-Cornell Medicine, New York, USA; 2 Department of Rheumatology, Instituto Nacional de Ciencias Médicas y Nutrición Salvador Zubirán, Mexico City, MEX; 3 Department of Pathology, Laboratorio Flemming, Morelos, MEX; 4 Department of Pathology, Instituto Nacional de Ciencias Médicas y Nutrición Salvador Zubirán, Mexico City, MEX

**Keywords:** vasculitis, splenectomy, granulomatosis with polyangiitis, sclerosing angiomatoid nodular transformation, spleen

## Abstract

We present an intriguing case involving a rare occurrence of sclerosing angiomatoid nodular transformation (SANT) in a 57-year-old woman with a history of granulomatosis with polyangiitis (GPA). Despite the extensive literature on SANT, its pathogenesis remains elusive. The patient, diagnosed with serum anti-proteinase 3 antineutrophil cytoplasmic antibody (PR3-ANCA)-positive GPA seven years earlier, exhibited a splenic lesion during imaging, leading to laparoscopic splenectomy due to severe abdominal pain. Microscopic analysis unveiled nodular structures with vascular elements surrounded by fibrosclerotic stroma and chronic inflammatory cells. This case raises questions about the interplay between SANT, GPA activity, and vascular damage. Hypotheses regarding SANT's origin, including its potential association with organized hematoma or alterations in splenic blood flow, are discussed. The uniqueness of this case lies in the coexistence of PR3-ANCA-positive GPA and SANT, suggesting a potential link between GPA activity, vascular damage, and SANT development. While causality remains uncertain, this report marks the first documented case of a patient with PR3-ANCA-positive GPA developing SANT. The findings prompt reflection on a potential common pathophysiological mechanism and underscore the importance of considering SANT in cases of splenic lesions associated with conditions causing alterations in splenic blood flow. This contribution serves as a valuable addition to the existing knowledge, urging further research and consideration of SANT in diagnostic scenarios involving splenic abnormalities.

## Introduction

Sclerosing angiomatoid nodular transformation (SANT) of the spleen is a rare benign splenic vascular lesion. At present its pathogenesis remains unclear. Since its initial description, there has been speculation regarding the possibility that this lesion could be a splenic hamartoma with sclerosis or a transformation of the red pulp due to an exaggerated stromal response. According to the latter hypothesis, the disruption of small vascular outflow tracts might lead to nodular changes, and these angiomatoid nodules could eventually progress to fibrous obliteration [1]. In the same way, it has been proposed that a subset of SANT may be related to the immunoglobulin (Ig)G4-related disease (IgG4-RD) [2] or the Epstein-Barr virus (EBV) [3]. This case report was conducted in accordance with the declaration of Helsinki of 1975 and obtained approval from the institutional bioethics committee.

## Case presentation

**Figure 1:**
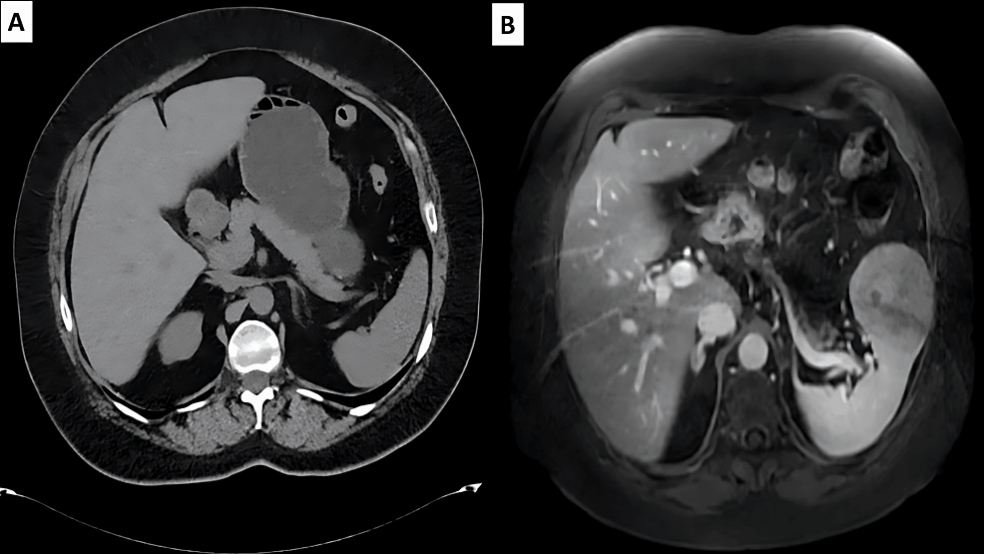
Computed tomography scan and magnetic resonance imaging at the splenic level (A) Computed tomography scan in 2012 in which a spleen of normal morphology and size was identified. (B) Magnetic resonance imaging in 2019 showing a well-circumscribed solid splenic lesion dependent on its anterolateral border of approximately 6.7 x 6.6 cm; the lesion is predominantly hypointense on T2 sequence with areas of central necrosis.

We present the case of a 57-year-old woman with a history of serum anti-proteinase 3 antineutrophil cytoplasmic antibody (PR3-ANCA)-positive granulomatosis with polyangiitis (GPA) diagnosed seven years before present history, with previous lacrimal gland, nasal, paranasal sinus, cutaneous, and lung involvement; at this time, a computerized tomography scan was performed without showing alterations in the spleen (Figure 1A). In February 2019, a chest computed tomography scan disclosed a splenic lesion in the lower pole, which was not observed in her initial workup for GPA. Three months later, an abdominal magnetic resonance imaging was performed, in which the same 6.7 x 6.6 cm lesion was identified (Figure 1B), with no increase in size; the differential diagnoses proposed by the imaging department were a SANT, less likely a hemangioma, without ruling out the possibility of a malignant neoplasm. However, due to the presence of oppressive abdominal pain with intensity of 7/10, a laparoscopic splenectomy was performed. 

**Figure 2:**
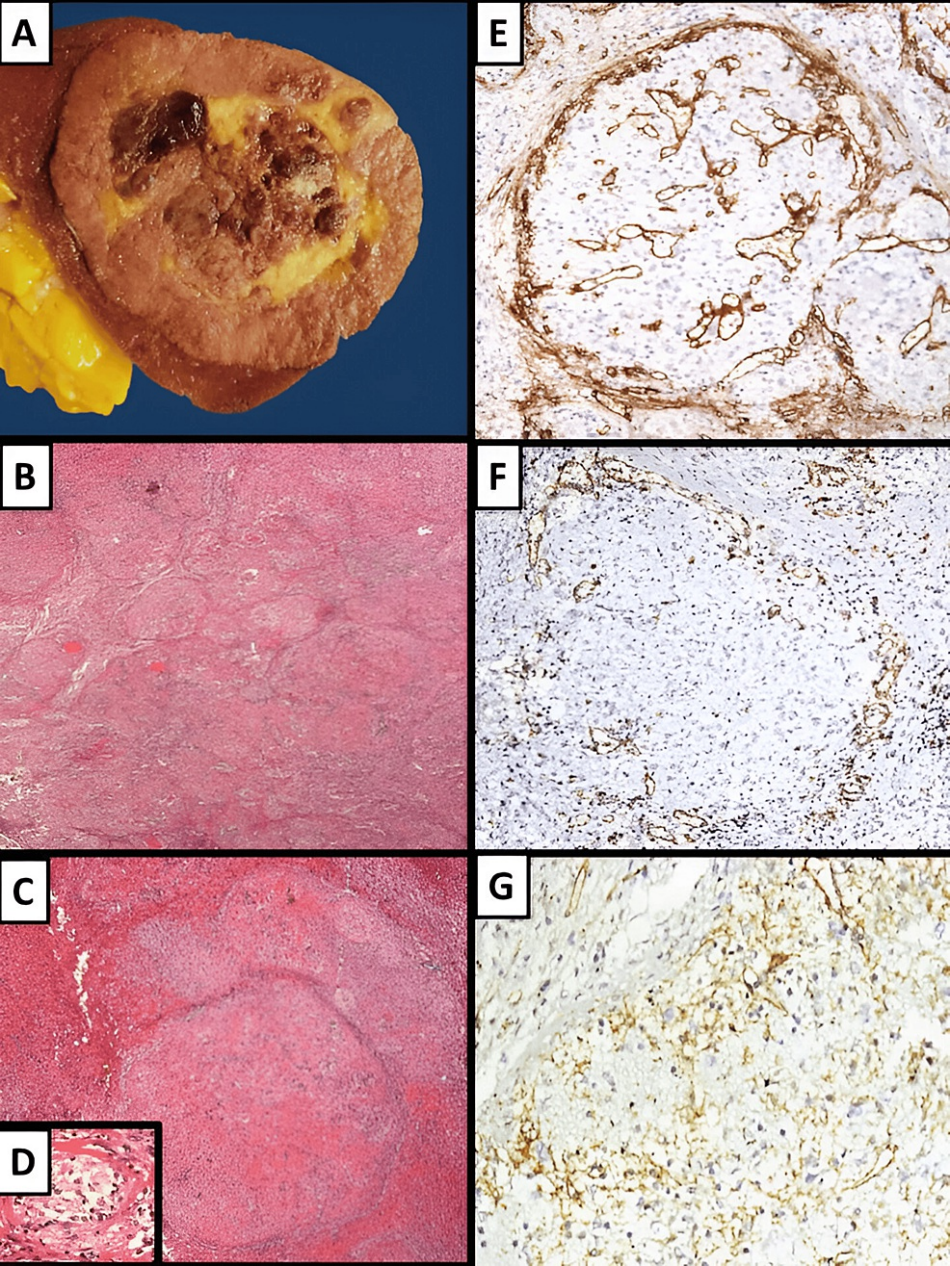
Macroscopic and microscopic findings of splenic lesion (A) Macroscopic examination of spleen revealed a well-circumscribed, nonencapsulated, and multinodular lesion. (B-D) Microscopic examination (hematoxylin and eosin stain) showed a multinodular lesion (B, 4x), composed of vascular spaces surrounded by fibrous connective tissue (C, 10x) and inflammatory cells (D, 40x). Immunohistochemical analysis documented positive expression of (E) CD34 immunostaining in the cord capillaries (10x), (F) CD8 immunostaining in the sinusoids (10x), and (G) CD31 immunostaining in the cord capillaries, sinusoids, and small veins (10x).

Macroscopically, a spleen of 261 grams was identified with a well-circumscribed, nonencapsulated, 6.0 x 4.3 cm multinodular lesion (Figure 2A). Microscopically, the lesion was formed by variably sized nodules with multiple vascular structures surround by a fibrosclerotic stroma with chronic inflammatory cells without necrosis (Figure 2B-2D). We did not identify morphological changes associated with GPA (fibrinoid necrosis, necrotizing arteritis, granulomas, or focal infarcts) in the splenic parenchyma not involved with the lesion. Few IgG4+ plasma cells were identified that corresponded to <30% of IgG+ plasma cells; also, Ziehl-Neelsen, CD30, ALK-1, CD21, CD23, and EBV-encoding region (EBER) in situ hybridization were negative (Figure 2E-2G). The patient had no clinical or radiological signs of IgG4-RD; thus, serology was not requested. She had no evidence of recurrence at four years' follow-up.

## Discussion

Even though ≈130 cases of SANT have been described [4], its pathogenesis remains unclear. Since the original description of SANT in 2004, the presence of a patient with von Willebrand disease and another with previous trauma has been described, hypothesizing that, in this clinical context, the SANT could have originated from an organized hematoma [1]. Likewise, recently a case has been published of a patient undergoing transcatheter arterial embolization for a splenic arterial aneurysm; this procedure resulted in a partial splenic infarction. Five years after this event, two splenic lesions measuring 35 x 34 mm (upper pole) and 15 x 12 mm (lower pole) were identified, which were later diagnosed as SANT [5]. In both publications, although it is not possible to confirm causality between the events, it is hypothesized that the alteration in splenic blood flow could have generated the development of SANT. 

In this same sense, the present case was documented in a patient with GPA, co-existence of entities that had not been previously described to our knowledge. This type of vasculitis is characterized by damage to small- to medium-caliber vessels and granuloma formation [6]. Although it is not possible to ensure that SANT will be generated as a consequence of GPA activity, some data that favor this association are the following: 1) At the time of the initial diagnosis of GPA (seven years prior to the diagnosis of SANT), the presence of lesions in the spleen were not identified by imaging studies. 2) It has been documented that up to 37% of patients with GPA suffer from splenic infarctions during their evolution [6]. All of these cases have been documented exclusively in patients like ours with GPA PR3-ANCA+ and not in GPA myeloperoxidase (MPO)-ANCA+ or ANCA-.

Taking into consideration that more than a third of PR3-ANCA+ GPA cases suffer from a splenic infarction during their evolution, we can hypothesize that either secondary to vascular damage induced by the disease in small or medium vessels or a potential splenic infarction will generate an alteration in the outflow of vascular tracts with subsequent nodular formation, fibrous progression, and formation of the SANT.

On the other hand, its potential association with IgG4-RD and EBV has been discussed [3], mainly due to the identification of IgG4+ plasma cells in the stroma [2]. In our case, we did not document the expression of EBER, and the presence of IgG4+ was scarce (4/42 IgG4/IgG), with no additional suspicious clinical or radiological data for this entity. Added to this, in the 2012 consensus on the pathology of IgG4-RD, this entity is classified within those with elevated IgG4+ plasma cells that fall outside of the IgG4-RD spectrum [7].

## Conclusions

In conclusion, we present to our knowledge the first case of a patient with PR3-ANCA+ GPA who developed a SANT in the evolution of the disease, with a potential common pathophysiological mechanism, and that, although it is not possible to demonstrate causality between both entities, this report can provide information for the development of animal models that test this pathophysiological mechanism and to consider the diagnostic possibility of SANT when there is a splenic lesion associated with a clinical condition that generates alterations in the splenic blood flow, such as splenic infarcts or vasculitis of small/medium vessels.
